# Celecoxib and shikonin loaded dissolving microneedle exert analgesic, anti-inflammatory and chondroprotective activity for osteoarthritis treatment

**DOI:** 10.3389/fcell.2026.1739175

**Published:** 2026-03-13

**Authors:** He Wang, Linsong Chen, Lei Zhou, Haifang Li, Xin Song, Jie Zhang, Hongmin Ma, Ping Ma

**Affiliations:** 1 Department of Anesthesiology, Qingdao University Affiliated Qingdao Third People’s Hospital, Qingdao, Shandong, China; 2 Department of Anesthesiology, Peking University People’s Hospital, Qingdao, Shandong, China; 3 Women and Children’s Hospital, Qingdao University, Qingdao, Shandong, China; 4 Department of Anesthesiology, Qingdao Eighth People’s Hospital, Qingdao, Shandong, China; 5 Pain Management, Qingdao University Affiliated Qingdao Third People’s Hospital, Qingdao, Shandong, China

**Keywords:** cartilage, celecoxib, dissolving microneedle, osteoarthritis, Percutaneous drug, shikonin

## Abstract

**Background:**

Osteoarthritis (OA) is a chronic degenerative disease that severely affects the physical function and quality of life of patients. Non-steroidal anti-inflammatory drugs (NSAIDs) are standard treatments for OA, alleviating joint pain and inflammation. Meanwhile, long-term oral administration and intra-articular injection will bring inevitable side effects. In this study, a dissolving microneedle composite drug delivery system co-loaded with celecoxib and shikonin (SKN-CXB@MN) was prepared, which can efficiently deliver drugs through the skin and exert therapeutic effects on OA from multiple targets including analgesia, anti-inflammation, and cartilage protection.

**Methods:**

The material properties of SKN-CXB@MN were assessed through experiments on morphology, mechanical properties, solubility, drug release performance, and skin recovery. The biocompatibility of SKN-CXB@MN was determined using CCK-8 assays and live-dead staining. The protective effect of SKN-CXB@MN on IL-1β-induced chondrocytes were investigated through *in vitro* anti-apoptosis assays. The analgesic, anti-inflammatory, and cartilage-protective effects of SKN-CXB@MN were evaluated by measuring the joint swelling degree, PWT, weight bearing of RL, histology, and immunohistochemical staining in the OA rat model.

**Results:**

Materials characterization reveals that SKN-CXB@MN possesses sufficient mechanical strength to penetrate the skin. Upon application, the microneedles dissolve completely and release 85% of CXB and 75% of SKN within 15 min . *In vitro* biocompatibility assays confirm that SKN-CXB@MN is non-cytotoxic to chondrocytes and does not affect cell proliferation. Anti-apoptosis experiments show that SKN-CXB@MN inhibits IL-1β-induced chondrocyte apoptosis. In an OA rat model, SKN-CXB@MN alleviates knee joint swelling and pain, reduces cartilage degeneration, decreases chondrocyte apoptosis, and suppresses the inflammatory response.

**Conclusion:**

The dual-drug loaded SKN-CXB@MN system facilitates targeted drug delivery, thereby reducing systemic side effects. The synergistic effects of Celecoxib (CXB) and Shikonin (SKN) not only substantially alleviate pain and inflammation but also retard cartilage degeneration by modulating chondrocyte apoptosis and extracellular matrix metabolism. These multifaceted mechanisms indicate that the SKN-CXB@MN formulation could be a promising therapeutic option for osteoarthritis management.

## 1 Introduction

Osteoarthritis (OA), a severe degenerative joint disease, can affect multiple components of the joint, including bones, cartilage, ligaments, and muscles, thereby impacting the entire joint structure. As the most common musculoskeletal disorder, the global prevalence of OA has risen substantially, from 4.8% (256 million) in 1990 to 7.6% (595 million) of the world’s population in 2020, posing a significant burden on global health (Tang et al., 2025; Yi et al., 2024; Courties et al., 2024).

OA involves cellular stress and extracellular matrix (ECM) degradation, often initiated by joint trauma. This condition disrupts joint tissue metabolism, leading to cartilage degradation, bone remodeling, osteophyte formation, and joint inflammation, ultimately impairing joint function and causing pain and disability. Despite OA’s prevalence and its significant burden on healthcare, treatment options remain limited. No non-surgical interventions exist to prevent, halt, or slow OA progression. Non-steroidal anti-inflammatory drugs (NSAIDs), the primary treatment, are available via topical, oral, or intra-articular routes. Prolonged oral use can lead to gastrointestinal bleeding and liver damage, while intra-articular injections require professional administration, limiting home care. NSAIDs alleviate symptoms but do not prevent cartilage damage progression and lose effectiveness in advanced disease stages. Thus, there is an urgent clinical need for a local drug delivery system that is efficient, convenient, provides analgesia, and addresses cartilage damage (Di Francesco et al., 2022; Cho et al., 2021).

Microneedles (MNs) represent a novel drug delivery technology that can physically penetrate the stratum corneum, creating microchannels that avoid blood vessels and nerves. This design makes microneedles a minimally invasive, painless, and efficient means of delivering drug components, combining the advantages of non-invasive transdermal and invasive injection drug delivery. Microneedle-based delivery can achieve high patient compliance for both short-term and long-term treatments, emerging as a promising approach for OA management (Wang J. et al., 2022; Li et al., 2025; Choupani et al., 2024; Li et al., 2024). Compared to traditional transdermal patches and oral medications, microneedles can significantly enhance transdermal drug delivery efficiency and circumvent the first-pass hepatic effect. With ongoing research, the application of microneedles has expanded to diverse areas, including dermatological treatment (Lv et al., 2025; Hou et al., 2025), insulin delivery for diabetes (Chellathurai et al., 2025), cancer therapy (Lin et al., 2025), ocular drug delivery (Gade et al., 2025; Choi et al., 2025), and vaccination (Driskill et al., 2025; Zou et al., 2025). Dissolving microneedles are typically fabricated by mixing biodegradable, biocompatible polymers with drug reagents. This single-step delivery method offers the advantages of high drug loading capacity, painless drug release, and rapid dissolution and diffusion of the encapsulated therapeutics (Xiang et al., 2025).

Celecoxib (CXB), a cyclooxygenase-2 (COX-2) inhibitor, is a widely used drug for relieving joint pain and inflammatory symptoms associated with conditions such as OA (Roda et al., 2025). To address the limitations of oral and injectable CXB administration, researchers have developed microneedle-based drug delivery systems. Wang et al. (Wang Q. et al., 2022) designed a microneedle array loaded with CXB nanocrystals, which improved the dissolution rate and bioavailability of the poorly soluble drug. Similarly, (Li et al., 2023) constructed a microemulsion-based dissolving microneedle co-loaded with CXB and α-linolenic acid, enhancing the treatment of OA through synergistic anti-inflammatory effects and efficient transdermal delivery. These microneedle systems achieve sustained drug release, overcoming the drawbacks of current administration methods. However, they do not prevent the progression of cartilage damage associated with OA.

The pathogenesis of OA is closely linked to ECM degradation and chondrocyte apoptosis in affected joints. Apoptotic cell death has been observed in OA cartilage, with the proportion ranging from less than 1% to approximately 20%, positively correlated with the severity of cartilage degeneration (Hwang and Kim, 2015; Werry et al., 2023). Therefore, anti-apoptotic therapies may offer a novel treatment approach for OA. Zicao, a traditional Chinese medicinal herb, contains various bioactive compounds, including naphthoquinones, benzoquinones, flavonoids, and polysaccharides. Shikonin (SKN), the main active ingredient, exhibits anti-inflammatory and antioxidant properties (Ye et al., 2025; Song et al., 2023; Yadav et al., 2022; Wang A. et al., 2022; Wang et al., 2024). Recent studies have demonstrated that SKN can inhibit the inflammatory response and chondrocyte apoptosis, thereby exerting a protective effect against OA in animal models and suggesting its potential as a therapeutic agent for OA (Li et al., 2015; Fu et al., 2016).

In this study, a dissolving microneedle composite drug delivery system co-loaded with CXB and SKN (SKN-CXB@MN) was prepared, which achieved efficient transdermal drug delivery and exerted therapeutic effects on OA from multiple targets, including analgesia, anti-inflammation, and cartilage protection. The morphology, dissolution test, drug release rate, mechanical properties, and skin recovery of SKN-CXB@MN were analyzed through materials science characterization experiments. The safety of SKN-CXB@MN for clinical application was verified by *in vitro* biocompatibility experiments. The therapeutic effect of SKN-CXB@MN on OA was evaluated by *in vitro* anti-apoptosis experiments and OA rat models.

## 2 Materials and methods

### 2.1 Materials and reagents

High-glucose DMEM medium, fetal bovine serum, streptomycin-penicillin, and DMSO were acquired from Thermo Fisher Scientific, USA. The Cell Counting Kit-8 (CCK-8) and Calcein-AM/PI Double Staining Kit were sourced from Dojindo Laboratories, Japan. Rat TNF-α, IL-1β, IL-6 ELISA Kit, One Step TUNEL Apoptosis Assay Kit, gelatin, and PVA were obtained from Beyotime Biotechnology, Shanghai. Sodium iodoacetate (MIA), shikonin, and celecoxib were procured from MedChemExpress (MCE), United States.

### 2.2 Animals

Forty-eight 6-week-old male rats weighing (220 ± 20) g were purchased from Beijing Vital River Laboratory Animal Technology Co., Ltd. This study was approved by the Experimental Animal Ethics Committee of Qingdao Third People’s Hospital. The experimental operations were strictly carried out in accordance with the specifications of the Guide for the Care and Use of Laboratory Animals. After 7 days of adaptive feeding under standard conditions, the rats were used for subsequent experimental operations, including testing the performance of MNs, extracting primary chondrocytes, establishing OA models, and microneedle treatment.

### 2.3 Preparation of CXB@MN and SKN-CXB@MN

Prepare a 20% (w/v) gelatin stock solution. Weigh CXB and SKN and dissolve them in DMSO respectively to prepare a 200 mg/mL CXB stock solution and a SKN stock solution. Mix the gelatin stock solution with the CXB stock solution to prepare a gelatin-CXB precursor solution. The final concentrations should be 10% (w/v) for gelatin and 500 μg/mL for CXB. Mix the gelatin stock solution, the CXB stock solution, and the SKN stock solution to prepare a gelatin-CXB-SKN precursor solution. The final concentrations of gelatin, CXB, and SKN are 10% (w/v), 500 μg/mL, and 500 μg/mL, respectively.

**FIGURE 1:**
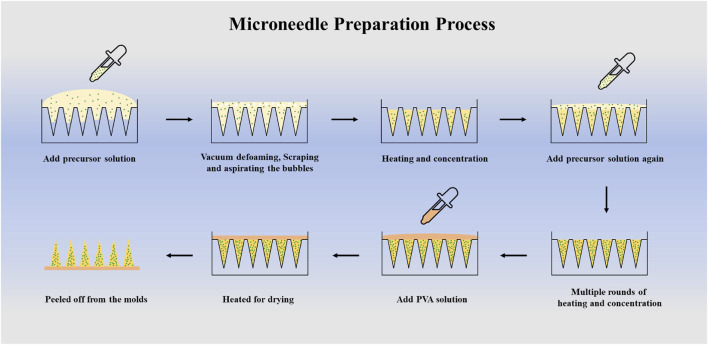
Schematic of microneedle preparation process.

The prepared gelatin-CXB and gelatin-CXB-SKN precursor solutions were carefully poured onto the microneedle mold, which was immediately transferred to a vacuum drying oven to remove entrapped bubbles. After vacuum treatment, the mold surface was scraped with a blade to remove residual surface bubbles. Subsequently, the molds were then heated and concentrated in a vacuum drying oven at 35 °C for 2–4 h. When the precursor solutions in the molds became viscous, the two precursor solutions were added dropwise respectively for multiple rounds of heating and concentration. Finally, a 20% (w/v) PVA solution was added to form the microneedle backing layer, and the molds were heated at 30 °C overnight for drying. After the backing layer was completely solidified, the microneedles were peeled off from the molds (Figure 1).

### 2.4 Characterization of CXB@MN and SKN-CXB@MN

#### 2.4.1 Morphological characterization

The morphological structures of MNs were observed using a digital camera (Nikon, Japan), and a scanning electron microscope (SEM, Philips XL-30, Netherlands).

#### 2.4.2 Mechanical properties

The mechanical strength of the microneedles was evaluated using a universal testing machine equipped with a flat stainless-steel probe (diameter: 25 mm). The microneedle patch was affixed horizontally to the testing platform with 3M double-sided adhesive tape. A flat-ended cylindrical probe was aligned vertically above the center of a single microneedle tip. The probe was aligned centrally over the tip of a microneedle and then moved downward at a constant rate of 0.1 mm/s. The fracture/buckling load and the corresponding force-displacement curve were recorded. The stiffness was calculated by extracting the slope from the start of the experiment to the maximum load.

#### 2.4.3 Penetration ability in the skin

To evaluate the transdermal performance of MNs, the MNs were pressed onto the surface of excised pig skin for 10 min. After removing the MNs, the micropores formed on the skin surface were observed and photographed. The skin tissue was fixed with 4% paraformaldehyde for 24 h, followed by dehydration, fixation, and embedding to prepare histological sections. The ability of MNs to penetrate the skin tissue was observed by Hematoxylin and eosin (HE) staining.

#### 2.4.4 *In vitro* dissolution properties

To evaluate the *in vitro* dissolution performance of the MNs, the MN tips were fluorescently labeled with rhodamine for visual assessment of their structural integrity. A 1.5% agarose hydrogel was prepared in PBS (pH 7.4). The hydrogel was allowed to solidify in a mold, forming a matrix sheet. The fluorescently labeled MNs were inserted into the agarose matrix sheet, and an *in vitro* dissolution experiment was conducted at 37 °C. The MNs were removed after 5, 10, and 15 min, respectively, and their dissolution was observed using a fluorescence microscope (TS2-FL, Nikon, Japan).

#### 2.4.5 *In vivo* dissolution properties

The dissolution properties *in vivo* of MNs were assessed. After the rats were anesthetized, their hair was shaved. MNs were pressed into the rat skin under a constant pressure. The MNs were removed at 5, 10, and 15 min respectively, and an optical microscope (TS100, Nikon, Japan) was used to observe and measure the remaining length of the needle tips.

#### 2.4.6 *In vitro* drug release

The MNs array was vertically inserted into the surface of the agarose matrix sheet, ensuring the needle tips were fully inserted. The device was placed in a 37 °C incubator, and samples of the agarose matrix sheet were collected at 0, 5, 15, and 30 min from the area where the microneedles were located. The samples were ground and then soaked and lysed in 2 mL of PBS for 30 min to allow the drug to be released into the solution. The undissolved precipitate was removed by centrifugal filtration, and the drug concentration was detected using ultraviolet spectrophotometry.

#### 2.4.7 Skin recovery assay

The skin recovery status following the application of MNs was evaluated. Rats were anesthetized, and the hair on the knee joint area was shaved and disinfected with 75% alcohol. MNs were applied to the skin of rats for 10 min and then removed. The treated areas were observed and photographed at 0, 1, and 2 h after the removal of the MNs.

### 2.5 Cell culture

Chondrocytes were isolated from the knee joint cartilage of SD rats. The cartilage was sectioned into fragments under 1 mm^3^ and washed with phosphate-buffered saline containing antibiotics. These fragments underwent digestion with type II collagenase in a 37 °C shaker for 4 h. The chondrocytes were collected and cultured in a 37 °C, 5% CO_2_ incubator for further cellular experiments (Chen et al., 2024).

### 2.6 Biocompatibility analysis

The cytotoxic effects of CXB@MN and SKN-CXB@MN on chondrocytes were assessed using the CCK-8 assay. CXB@MN and SKN-CXB@MN were individually dissolved in culture medium, filtered through a 0.22 μm membrane, and supplemented with 5% fetal bovine serum and 1% streptomycin-penicillin antibiotics to prepare the treatment media. Chondrocytes were seeded in a 96-well plate at a density of 5 × 10^3^ cells per well and cultured overnight. The culture medium was then replaced with the prepared treatment media, and the cells were cultured for 1, 3, and 5 d. Subsequently, the CCK-8 reagent was added, and the cells were incubated for 2 h. The absorbance of each well was measured at 450 nm using a microplate reader (Multiskan FC, Thermo Fisher, United States) (n = 5).

The viability of chondrocytes exposed to CXB@MN and SKN-CXB@MN was assessed through live/dead cell staining. Chondrocytes were seeded at a density of 4 × 10^4^ cells per well in a 24-well plate containing cell climbing sheets and cultured until 80% confluent. The medium was then replaced with above-mentioned medium containing CXB@MN or SKN-CXB@MN, and the cells were incubated for 24 h. Following the manufacturer’s protocol, a cell staining solution was added, and the plates were incubated at 37 °C in the dark for 15 min. After PBS washing, live and dead cells were simultaneously imaged using a fluorescence microscope (TS2-FL, Nikon, Japan), with live cells appearing green and dead cells appearing red. Quantitative analysis was performed using ImageJ software.

### 2.7 *In vitro* anti-apoptosis evaluation

The chondrocyte apoptosis was assessed using the One Step TUNEL Apoptosis Assay Kit. Chondrocytes were seeded in 6-well plates and cultured until they reached 80% confluence. The cells were then treated with IL-1β (10 ng/mL) or a combination of IL-1β and CXB@MN and SKN-CXB@MN for 24 h. Subsequently, the cells were washed with PBS, fixed with 4% paraformaldehyde for 30 min, and washed with PBS again. The cells were permeabilized with PBS containing 0.3% Triton X-100 at room temperature for 5 min and washed twice with PBS. The TUNEL detection solution was prepared according to the manufacturer’s instructions and added to each well (100 μL per well). The cells were incubated at 37 °C in the dark for 60 min, washed three times with PBS, and the nuclei were stained with DAPI. Fluorescence microscopy was used for observation and imaging, with the excitation wavelength range set at 450–500 nm and the emission wavelength range at 515–565 nm.

### 2.8 OA rat model and treatment

OA was induced by injecting MIA into the right knee joint of SD rats. As previous reported (Zhou et al., 2021), after anesthetizing the rats, 50 μL of 40 mg/mL MIA solution was injected into the right knee joint cavity, and the joint was flexed and extended repeatedly for 15 min to ensure the full diffusion of the MIA solution. The remaining rats were injected with an equal volume of normal saline. After 14 days, the rats injected with normal saline served as the control group. The rats injected with MIA were randomly divided into the model group, CXB@MN group, and SKN-CXB@MN group (n = 10). Rats in the CXB@MN (with 2 mg/mL CXB) and SKN-CXB@MN group (with 1mg/mLCXB and 1 mg/mL SKN) received microneedle treatment at the right knee joint every other day, while the control group and model group received no treatment. At 29th day, the rats were sacrificed for subsequent analysis.

### 2.9 Joint swelling measurement

The therapeutic efficacy of the drug treatment in OA rats was assessed by quantifying the degree of joint swelling. At 0, 7, 14, 21, and 28 days, the widths of the right knee joint’s tibial plates were measured bilaterally using a vernier caliper. The change in knee joint width (ΔW) was calculated as: $\Delta W=d_{T}-d_{0}$

Where d_T_ represents the width at the given timepoint and d_0_ the initial width. This metric served as an indicator of the extent of knee joint swelling.

### 2.10 PWT

The paw withdrawal threshold (PWT) was used to evaluate the pain sensitivity of the hind paws of rats using the von Frey pain threshold detector (BIO-EVF4-S, BIOSEB, France). Rats were placed on a metal grid rack and allowed to acclimate to the environment for 1 h. The experimental environment was ensured to be quiet to minimize interference with the animals. A suitable von Frey filament was gently applied to the plantar surface of the rat’s hind paw from beneath the grid, and the minimum force required to elicit a positive paw withdrawal response was recorded as the PWT. The test was repeated 5 times with an interval of 2 min between each test to ensure that the animals returned to the baseline state, and the average value was taken as the final result.

### 2.11 Weight bearing of RL

The weight distribution ratio between the left hind limb (LL) and right hind limb (RL) of rats was assessed to evaluate pain in the right hind limb using a bipedal balance analgesia meter (BIO-SWB-TOUCH-R, BIOSEB, France). Rats were placed in the inclined experimental chamber, with their hind paws positioned on independent force-measuring platforms. Each rat was tested five times, and the average value was calculated. The results were expressed as the percentage of weight-bearing, calculated as:

$$\text{Weight }\text{bearing }\text{of }\text{RL }\left(\%\right)=\frac{\text{weight}\;\text{bearing}\;\text{of}\;\text{RL}}{\text{weight}\;\text{bearing}\;\text{of}\;\text{RL}+\text{weight}\;\text{bearing}\;\text{of}\;\text{LL}}\times 100\%$$

### 2.12 Inflammatory factors in rat serum

Blood samples were collected from the orbital sinus of rats at 29th day. The whole blood was allowed to clot at room temperature for 2 h, then centrifuged at 4 °C and 2000 g for 10 min to separate the serum. The serum was collected and stored on ice for subsequent analysis. Levels of tumor necrosis factor-α (TNF-α), interleukin-1β (IL-1β), and interleukin-6 (IL-6) in the serum were measured according to the provided instructions.

### 2.13 Gross observation and histological examination

Remove the excess muscle tissue, the rat knee joint samples were photographed and observed the changes in joint structure.

The knee joints were fixed in 4% paraformaldehyde for 24 h, followed by decalcification at 37 °C until a pin could easily penetrate the tissue, indicating the endpoint of decalcification. The samples were then rinsed under running water. After dehydration, fixation, and embedding, the samples were serially sectioned into 5-μm-thick slices. HE, and safranin O-fast green (SO-FG) staining were employed to evaluate the tissue structure and cytological changes in the knee joints. The degree of cartilage degeneration was assessed using a histopathological scoring system (OARSI scoring standard) based on the extent of cartilage injury and calcification.

In addition, immunohistochemical staining of type II collagen (COL-2), and matrix metallopeptidase 13 (MMP-13) was used to further assess the progression of osteoarthritis, and cartilage degeneration, and quantitative analysis was performed using ImageJ software. All histological assessments were carried out employing a double-blind observation method.

### 2.14 Statistical analysis

Statistical analyses were conducted using GraphPad Prism 9.5. Data are presented as mean ± standard deviation. The One-way ANOVA was used to evaluate statistical differences, with *p* < 0.05 indicating statistical significance. All outcome assessments were performed with blinding and randomization. ****p < 0.0001, ***p < 0.001, **p < 0.01, *p < 0.05.

## 3 Results

### 3.1 Characterization analysis

#### 3.1.1 Morphological characterization

**FIGURE 2:**
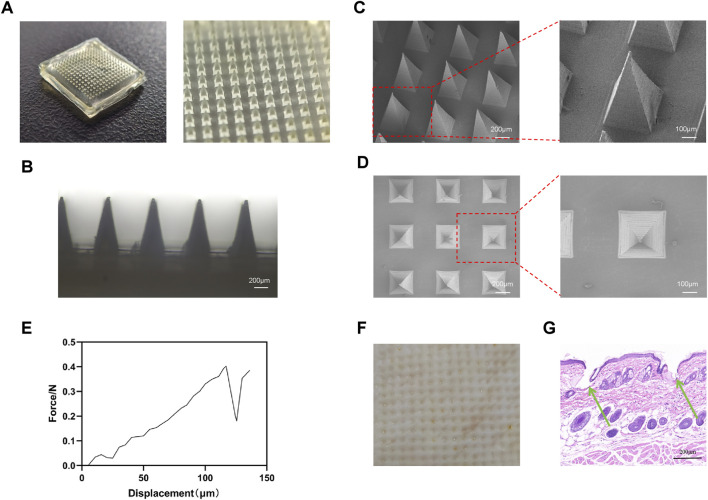
Morphological, mechanical characterization of MNs and skin penetration assay. **(A)** Image of digital microscope. **(B)** Image of optical microscopy. **(C,D)** Images of SEM. **(E)** Mechanical test curve of MNs. **(F)** Gross observation of skin penetration assay. **(G)** HE staining of skin penetration assay. The green arrows indicate the conical depressions in the skin tissue caused by the insertion of MNs.

In this study, CXB@MN and SKN-CXB@MN were successfully prepared. Digital camera images, and SEM images showed that the MNs arrays consisted of 225 microneedles (15 × 15 array). The height of sharp tips was about 800 μm and these MNs had uniform morphology and size, meeting the requirements for transdermal drug delivery (Figures 2A–D).

#### 3.1.2 Mechanical property and skin penetration assay

The mechanical property of MNs is a crucial factor for their ability to effectively penetrate the stratum corneum and deliver drugs to the subcutaneous tissue. The mechanical test indicated that each microneedle can withstand a force of approximately 0.4 N before bending or breaking, significantly exceeding the 0.1 N threshold force required for a single needle to penetrate the skin (Zhang et al., 2023). (Figure 2E) The above mechanical property of MNs ensures that the microneedle patch can achieve successful drug delivery.

Stable and reliable skin penetration ability is a key indicator for the biomedical application of microneedles. Visual observation revealed that the MNs can smoothly pierce the skin, forming a regular sequence of micropores (Figure 2F). The result of HE staining showed that the MNs penetrated the stratum corneum, and the conical depressions formed in the skin tissue perfectly matched the shape of the microneedles (Figure 2G).

These results demonstrated that the prepared MNs possessed excellent mechanical properties and skin puncture ability, supporting their potential for further research and development.

#### 3.1.3 Dissolution characterization

**FIGURE 3:**
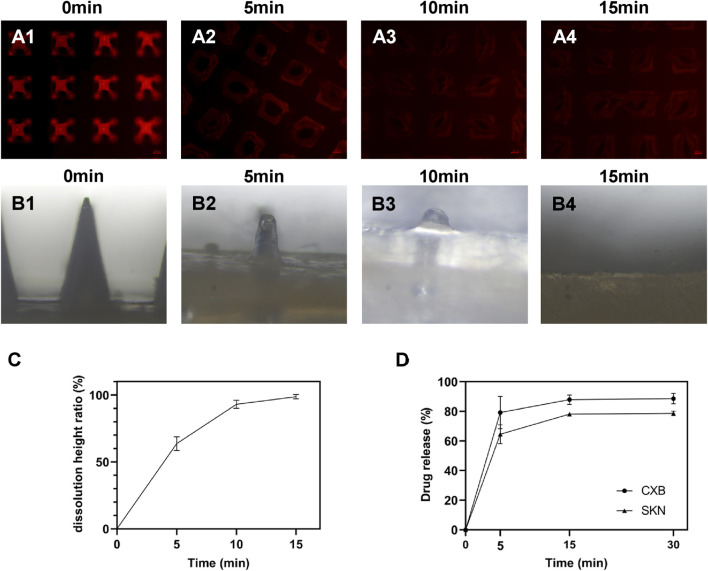
Dissolution and drug release of MNs. Images of dissolution of MNs *in vitro* after **(A1)** 0 min, **(A2)** 5 min, **(A3)** 10 min, and **(A4)** 15 min. Images of dissolution of MNs *in vivo* after **(B1)** 0 min, **(B2)** 5 min, **(B3)** 10 min, and **(B4)** 15 min. **(C)** Dissolution curve of MNs, n = 3. **(D)** Drug release of MNs, n = 3.

The dissolution characteristics of MNs play a critical role in subcutaneous drug delivery. An ideal dissolving MNs should dissolve rapidly and release the drug after piercing the skin. Firstly, the *in vitro* dissolution properties of MNs were evaluated using the agarose matrix sheet to simulate the penetration ability of the skin at 37 °C. The results showed that the MNs were completely dissolved in 15 min (Figures 3A1–A4).

To evaluate the *in vivo* dissolution properties of MNs, the MNs were applied to the skin and removed after 5, 10, and 15 min respectively. The height of the remaining MNs was observed and recorded. The results showed that the MNs array began to dissolve and the needle tips gradually became blunt. At 5 min, 65% of the MNs had completed dissolution; and at 15 min the MNs were completely dissolved (Figures 3B1–B4,C).

To sum up, MNs with rapid dissolution properties were successfully prepared, which can achieve good drug release and thus achieve therapeutic effects. Meanwhile, the MNs were convenient to use, which helped to shorten the treatment time and improved patient compliance.

#### 3.1.4 Skin recovery evaluation

Biocompatibility and non-irritation are essential prerequisites for the application of MNs to the human body. The biocompatibility of MNs was evaluated by assessing the skin recovery status after MNs insertion. MNs were applied to the skin of rats and removed after 10 min, and the skin recovery of the rats was observed. As shown in Supplementary Figures S1A–C, immediately after the removal of the microneedles, regular and orderly micropores were visible in both groups. The micropores gradually disappeared over time, and almost no micropores could be seen after 1 h. No obvious irritation reactions, such as swelling and erythema, occurred during the whole observation period. The results indicated that MNs have good biocompatibility and cause no irritation to the skin.

#### 3.1.5 Drug release *in vitro*

In the entire SKN-CXB@MN array, the content of CXB was 211.5 ± 8.8 μg, and the content of SKN was 209.4 ± 12.9 μg (n = 3). The results presented that demonstrate the rapid and extensive release kinetics of the two drug compounds from the microneedle delivery system. Specifically, the cumulative release of CXB reached 79% within 5 min and 88% within 15 min. Similarly, the cumulative release of SKN attained 65% at 5 min and 78% at 15 min (Figure 3D). These findings suggested that the microneedle tips can effectively penetrate the stratum corneum barrier of the skin, thereby facilitating the rapid delivery and onset of the pharmacological actions of the encapsulated drugs.

### 3.2 Biocompatibility analysis

**FIGURE 4:**
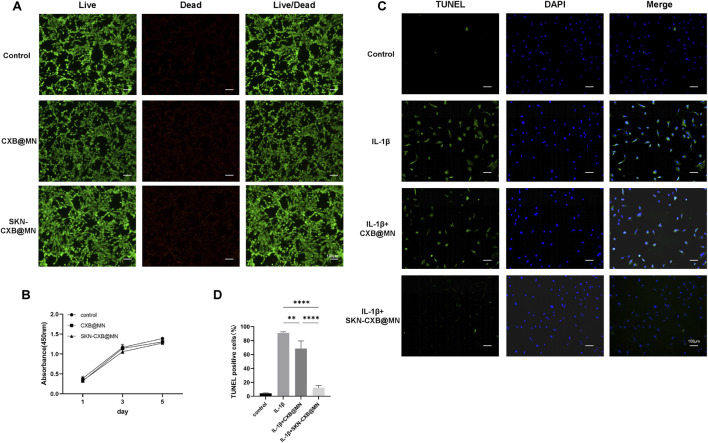
Biocompatibility analysis and anti-apoptosis evaluation. **(A)** Images of Live/dead staining. **(B)** Cell proliferation curve by CCK-8 assay, n = 5. **(C)** Images of TUNEL staining exposed to IL-1β. **(D)** Quantitative analysis of TUNEL staining, n = 3. ****p < 0.0001, **p < 0.01.

According to the results of live/dead staining, after co-culturing the 2 MNs with cells for 24 h, the chondrocytes grew well, the ratio of live cells was over 90%, and there was no statistical difference compared with the blank group, which confirming that both groups of MNs did not affect the growth viability of chondrocytes (Figure 4A; Supplementary Figure S1D).

The impact of CXB@MN and SKN-CXB@MN on chondrocyte proliferation and viability was assessed using CCK-8 assays and live/dead staining. As shown, by observing the cell proliferation curves at 1, 3, and 5 days, the cell proliferation rates of the CXB@MN and SKN-CXB@MN groups were slightly lower than those of the blank group, but there was no statistical significance, indicating that MNs had no toxic effect on cells (Figure 4B).

The above results indicated that CXB@MN and SKN-CXB@MN have good biocompatibility, which can be used for further *in vivo* experiments and clinical applications.

### 3.3 Anti-apoptotic effect

The TUNEL staining was employed to assess the preventive effects of CXB@MN and SKN-CXB@MN on IL-1β-induced chondrocyte apoptosis. The results of TUNEL staining showed that compared with the blank control, the apoptosis of chondrocytes was significantly increased after 24 h of treatment with IL-1β, which indicating that IL-1β could induce chondrocyte apoptosis. Compared with the IL-1β treatment group, the apoptosis rate of chondrocytes treated with the SKN-CXB@MN was significantly decreased, and the apoptosis rate of chondrocytes treated with the CXB@MN also showed a decreasing trend (Figures 4C,D). The results demonstrated that both CXB and SKN could inhibit IL-1β-induced chondrocyte apoptosis, with a more pronounced anti-apoptotic effect when the two acted in combination.

### 3.4 Efficacy of MNs on OA

#### 3.4.1 Joint swelling measurement

**FIGURE 5:**
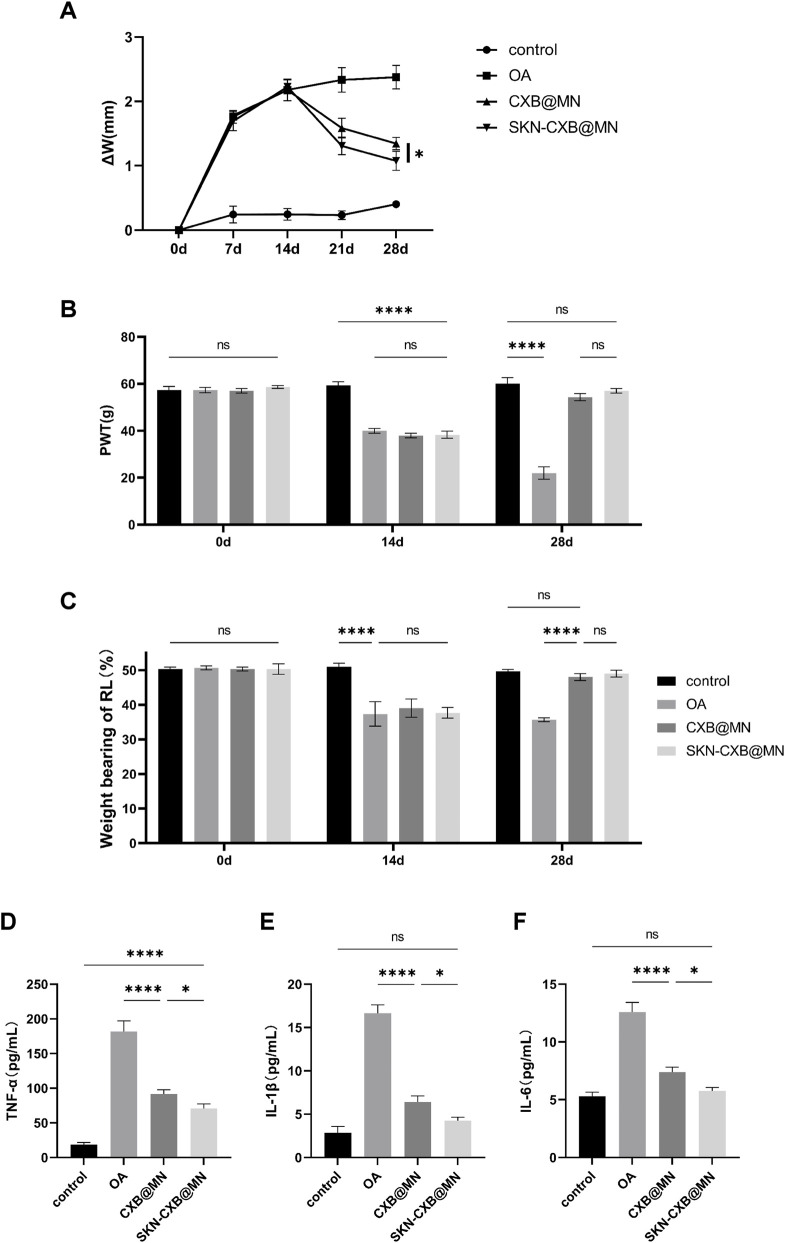
**(A)** The changes of knee joint width at 0, 7, 14, 21, and 28 days. The changes in **(B)** PWT and **(C)** weight bearing of RL of different groups at 0, 14, and 28d. Concentration of **(D)** TNF-α, **(E)** IL-1β and **(F)** IL-6 in rat serum after 14d of treatments. ****p < 0.0001, ***p < 0.001, **p < 0.01, *p < 0.05.

The MIA-induced OA model is a well-established and widely utilized preclinical model, exhibiting pathological features and pharmacological properties highly analogous to human OA. The right knee joint of rats in the MIA-induced OA model progressively swelled over time. At day 14, the right knee joint of OA model rats displayed significant swelling compared to the control group, indicating successful model establishment. Following the application of MNs to the right knee joint, both CXB@MN and SKN-CXB@MN demonstrated a downward trend in swelling, with a more pronounced decline observed in the SKN-CXB@MN group. A slight reduction in swelling was also noted in the OA group, potentially attributed to the body’s inherent self-repair mechanisms (Figure 5A). These findings suggested that both CXB@MN and SKN-CXB@MN possessed anti-inflammatory properties to effectively mitigate knee joint swelling.

#### 3.4.2 Changes in the pain threshold indexes in rats

Pain is a typical symptom of OA. Therefore, one of the goals of OA treatment drugs is to relieve knee joint pain. As shown, at day 14 both the PWT and the weight bearing of RL of OA model decreased significantly, indicating that pain occurred in the right knee joint of the rats and the modeling was successful. After treatment with MNs, the PWT and the weight bearing of RL in the CXB@MN and SKN-CXB@MN groups were significantly improved. There was no statistical significance between the two groups. The results indicated that both CXB@MN and SKN-CXB@MN had good analgesic effects (Figures 5B,C).

#### 3.4.3 Determination of inflammatory factors in rat serum

To evaluate the *in vivo* anti-inflammatory effects of CXB@MN and SKN-CXB@MN, the levels of TNF-α, IL-1β, and IL-6 in the serum were measured. The results showed that the levels of inflammatory factors in the serum of rats in the OA model were significantly increased. The levels of TNF-α, IL-1β, and IL-6 in the CXB@MN and SKN-CXB@MN groups were significantly decreased (Figures 5D–F).

#### 3.4.4 Gross observation

**FIGURE 6:**
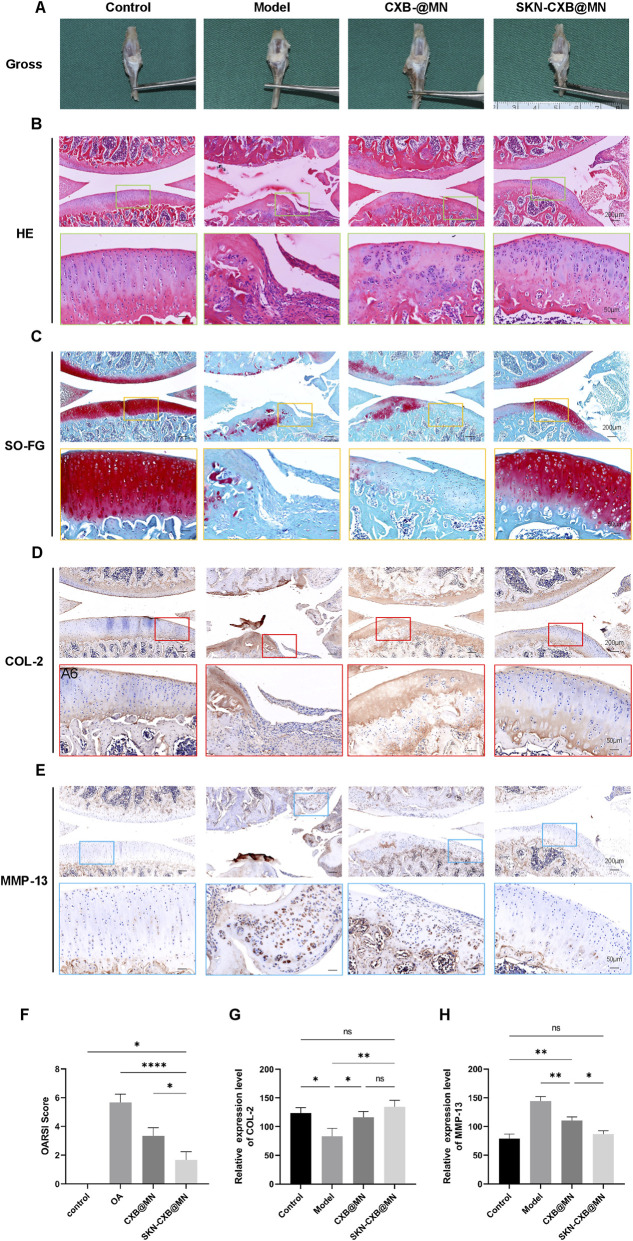
Gross observation and Histological evaluation of OA model. **(A)** Image of gross observation of rats’ knee joints. Image of **(B)** HE, and **(C)** SO-FG staining of rats’ knee joints. Immunohistochemical staining image of **(D)** COL-2, and **(E)** MMP-13 of rats’ knee joints. **(F)** The OARSI score, n = 3. **(G)** Quantitative analysis of COL-2 immunohistochemical staining, n = 3. **(H)** Quantitative analysis of MMP-13 immunohistochemical staining, n = 3. ****p < 0.0001, **p < 0.01, *p < 0.05.

On the 29th day post-modeling, rat sacrifices and sample collections were conducted to assess gross anatomical changes in the knee joints. The OA group exhibited classic osteoarthritis features: an enlarged right knee joint cavity, full-thickness cartilage loss exposing subchondral bone, thickened synovium, and joint effusion. After 14 days of treatment, the CXB@MN group showed an enlarged joint cavity with a dull, rough cartilage surface and localized cartilage degeneration and thinning, though with milder damage compared to the OA group. In contrast, the SKN-CXB@MN group displayed no significant anatomical changes or articular cartilage damage (Figure 6A).

#### 3.4.5 Histological evaluation

The histological structure, cytological changes, and ECM degradation of rat knee joint cartilage were evaluated by HE, SO-FG, and Masson staining combined with OARIS scoring. The results of HE staining showed that in the OA group, the normal histological structure of the joints was lost, with severe cartilage damage, complete absence of cartilage at the femoral end, and only a small amount of cartilage tissue remaining in the tibial segment. Few chondrocytes were observed, and there was a large amount of inflammatory cell infiltration. After MNs treatment, cartilage damage showed varying degrees of improvement, and inflammatory cells were markedly reduced. In the CXB@MN group, cartilage damage significantly decreased, and the histological structure remained relatively intact. However, there was a substantial amount of chondrocyte apoptosis and a disrupted tidemark, indicating the disease’s progression. The SKN-CXB@MN group showed the most significant therapeutic effect, with a complete joint histological structure, a relatively clear and continuous tidemark, and relatively less chondrocyte apoptosis. No inflammatory cell infiltration was observed in either the CXB@MN group or the SKN-CXB@MN group (Figure 6B). The results of the OARSI score showed that the score of the SKN-CXB@MN group was significantly lower than those of the OA and CXB@MN groups (Figure 6F).

The degradation of the cartilage ECM was further assessed using SO-FG staining. As shown in the figure, the OA group experienced almost complete depletion of proteoglycans, with significant ECM degradation. The CXB@MN group showed relatively more matrix degradation. In contrast, the SKN-CXB@MN group retained more proteoglycans, and the matrix degradation was comparatively less (Figure 6C).

The findings demonstrated that both the CXB@MN and SKN-CXB@MN groups exhibited anti-inflammatory properties. Notably, the SKN-CXB@MN group was able to inhibit cartilage degradation and delay the further advancement of osteoarthritis.

#### 3.4.6 Immunohistochemical evaluation

Immunohistochemical staining was used to evaluate the expression of COL-2 and MMP-13 in articular cartilage, aiming to elucidate the mechanism of osteoarthritis treatment in the CXB@MN and SKN-CXB@MN groups. Notably, the expression of COL-2 in the CXB@MN and SKN-CXB@MN groups was significantly higher than that in the OA group, and the expression in the SKN-CXB@MN group was closer to that in normal tissues. Moreover, compared with the control and SKN-CXB@MN groups, the expression of MMP-13 was significantly increased in the OA and CXB@MN groups (Figures 6D,E,G,H).

## 4 Discussion

The age-standardized disability-adjusted rate of OA globally increased by 9.5% between 1990 and 2020, establishing it as the seventh leading cause of motor dysfunction in individuals aged 70 and above (GBD, 2021 Osteoarthritis Collaborators, 2023). The knee joint is the most commonly affected site among this population. Current OA treatments fall into three categories: non-pharmacological, pharmacological, and surgical. Non-pharmacological approaches, like exercise and weight loss, alleviate symptoms but do not facilitate joint structure repair. Pharmacological strategies, primarily involving NSAIDs and glucocorticoids, are hindered by NSAIDs’ risks of hepatotoxicity, nephrotoxicity, gastrointestinal issues, and cardiovascular complications, while glucocorticoid injections, despite localized effects, necessitate professional administration and carry risks of pain, tissue damage, and infection, reducing patient compliance. Surgical options, such as joint replacement, significantly enhance function in advanced OA but entail high trauma, cost, postoperative complications, and limited prosthesis lifespan. Overall, while current treatments mitigate pain and inflammation, they offer only temporary relief and fail to regenerate cartilage or halt disease progression. The systemic side effects of drugs and the invasiveness and cost of surgery limit their clinical utility (Honvo et al., 2025; Moseng et al., 2024; Allen et al., 2025; Postler et al., 2023; Ciaffi et al., 2024). Therefore, developing novel therapeutic strategies that enhance targeted drug delivery efficiency while minimizing systemic adverse reactions is urgently needed to improve the long-term management of OA.

In this study, a dissolving microneedle co-loaded with CXB and SKN was innovatively prepared. First, SKN-CXB@MN can achieve targeted drug delivery, avoiding the drawbacks of oral and intra-articular drug administration. SKN-CXB@MN enables transdermal drug delivery by breaking through the physical barrier of the skin. It has sufficient mechanical strength and a uniform needle structure. The needle tip with a height of 800 μm can penetrate the stratum corneum and reach the epidermis without damaging the nerves and blood vessels in the dermis. Meanwhile, it causes almost no pain, effectively improving patient compliance. Second, SKN-CXB@MN has high drug delivery efficiency. The needle tips can completely dissolve within 15 min under physiological conditions, and 88% of CXB and 78% of SKN can be rapidly delivered to the joint cavity within 15 min to exert their effects. In addition, SKN-CXB@MN has good biocompatibility. The puncture marks almost disappear within 1 h after application to the skin, and it does not cause irritation.

Recent preclinical and clinical evidence underscores inflammation as a central factor in OA development. This inflammation is a complex process involving multiple cell and tissue types both within and beyond the joint. While inflammation is vital for tissue repair and restoring homeostasis, its unchecked and dysregulated form underpins chronic inflammatory diseases. Synovitis is a hallmark of joint inflammation, with articular cartilage, meniscus, and subchondral bone also engaging in the inflammatory response (Knights et al., 2023). Pain, a primary clinical feature and patient concern in osteoarthritis, is increasingly linked to inflammation. There is a strong correlation between synovitis severity and pain, highlighting inflammation as a key target for alleviating pain symptoms (Geraghty et al., 2021; Baker et al., 2010; Gómez-Aris et al., 2019; Neogi, 2013). In this study, *in vivo* experiments demonstrated that SKN-CXB@MN and CXB@MN effectively reduced knee joint swelling and pain in osteoarthritis rats.

Cartilage degradation is one of the prominent features of OA. The processes of chondrocyte growth, differentiation, and apoptosis are strictly regulated to maintain dynamic equilibrium, thereby achieving a benign cycle of cartilage matrix synthesis and degradation under normal physiological conditions. However, under OA stimulation, chondrocytes transform into catabolic cells, secreting matrix-degrading enzymes such as matrix metalloproteinases (MMPs) and a disintegrin and metalloproteinase with thrombospondin motifs (ADAMTSs). Meanwhile, they lose the ability to maintain survival and undergo apoptosis, ultimately leading to articular cartilage degeneration (Xiao et al., 2023; Ma et al., 2024). Articular cartilage degeneration induced by chondrocyte apoptosis plays a crucial role in the pathogenesis of OA. IL-1β, as an important pro-inflammatory cytokine, has been proven to induce chondrocyte apoptosis in both *in vivo* and *in vitro* environments, resulting in the degradation of the chondrocyte ECM and promoting the progression of OA (Wang et al., 2019). Therefore, inhibiting chondrocyte apoptosis is of great significance in the treatment of OA. Fu et al. (Fu et al., 2016) found that SKN could inhibit the inflammatory response and chondrocyte apoptosis by regulating the PI3K/Akt signaling pathway. In this study, an *in vitro* cell model of OA was constructed by inducing chondrocytes with IL-1β. A large number of cells in the IL-1β group underwent apoptosis, while the number of apoptotic cells in the SKN-CXB@MN group was significantly reduced, further confirming previous reports.

Additionally, upregulated cytokines in OA can promote the upregulation of COX-2 and inducible nitric oxide synthase (iNOS) expression, which in turn increases the expression of MMPs and ADAMTSs. Enzymes in the MMPs family, such as MMP-13, mainly degrade COL-2 and proteoglycans and inhibit their synthesis, leading to a large loss of the ECM. As a result, chondrocytes lose their normal living environment, indirectly triggering chondrocyte apoptosis (Xiao et al., 2024; Motta et al., 2023). CXB, a nonsteroidal anti-inflammatory drug, selectively inhibits cyclooxygenase-2 and is used to alleviate pain and inflammation in patients with knee osteoarthritis. Haartmans et al. (2022) investigated CXB potential chondroprotective effects on human articular cartilage explants and in an *in vivo* osteoarthritis rat model. Their findings revealed a significant reduction in MMP-13 levels following CXB treatment in human cartilage explants. Additionally, cartilage degeneration was notably diminished in the *in vivo* osteoarthritis knee rat model. In this study, the immunohistochemical staining results of MMP-13 and COL-2 in the OA rat model indicated that SKN-CXB@MN could inhibit the expression of MMP-13, reduce ECM degradation, and further decrease chondrocyte apoptosis. These results all suggest that SKN-CXB@MN can exert a chondroprotective effect by inhibiting chondrocyte apoptosis.

## 5 Conclusion

The dual-drug loaded SKN-CXB@MN represents a promising multimodal therapeutic approach for OA. CXB, a selective COX-2 inhibitor, is a widely used oral treatment for OA. The addition of SKN, a natural compound, addresses the limitations of traditional non-steroidal anti-inflammatory drugs. The synergistic combination of CXB and SKN not only significantly alleviates pain and inflammation, but also delays the progression of cartilage degeneration by regulating chondrocyte apoptosis and extracellular matrix metabolism. This multifaceted mechanism of action suggests that the SKN-CXB@MN formulation may offer a valuable therapeutic option for the management of OA.

## Data Availability

The original contributions presented in the study are included in the article/Supplementary Material, further inquiries can be directed to the corresponding authors.
