# Supplementary material for: Celecoxib and shikonin loaded dissolving microneedle exert analgesic, anti-inflammatory and chondroprotective activity for osteoarthritis treatment
Source: Front Cell Dev Biol. 2026 Mar 13;14:1739175. doi: 10.3389/fcell.2026.1739175 (PMC13021894; doi:10.3389/fcell.2026.1739175)
Supplement: Supplementary file 1 [file DataSheet1.docx]

Supplementary Material

# Supplementary Figures and Tables

## Supplementary Figures

**
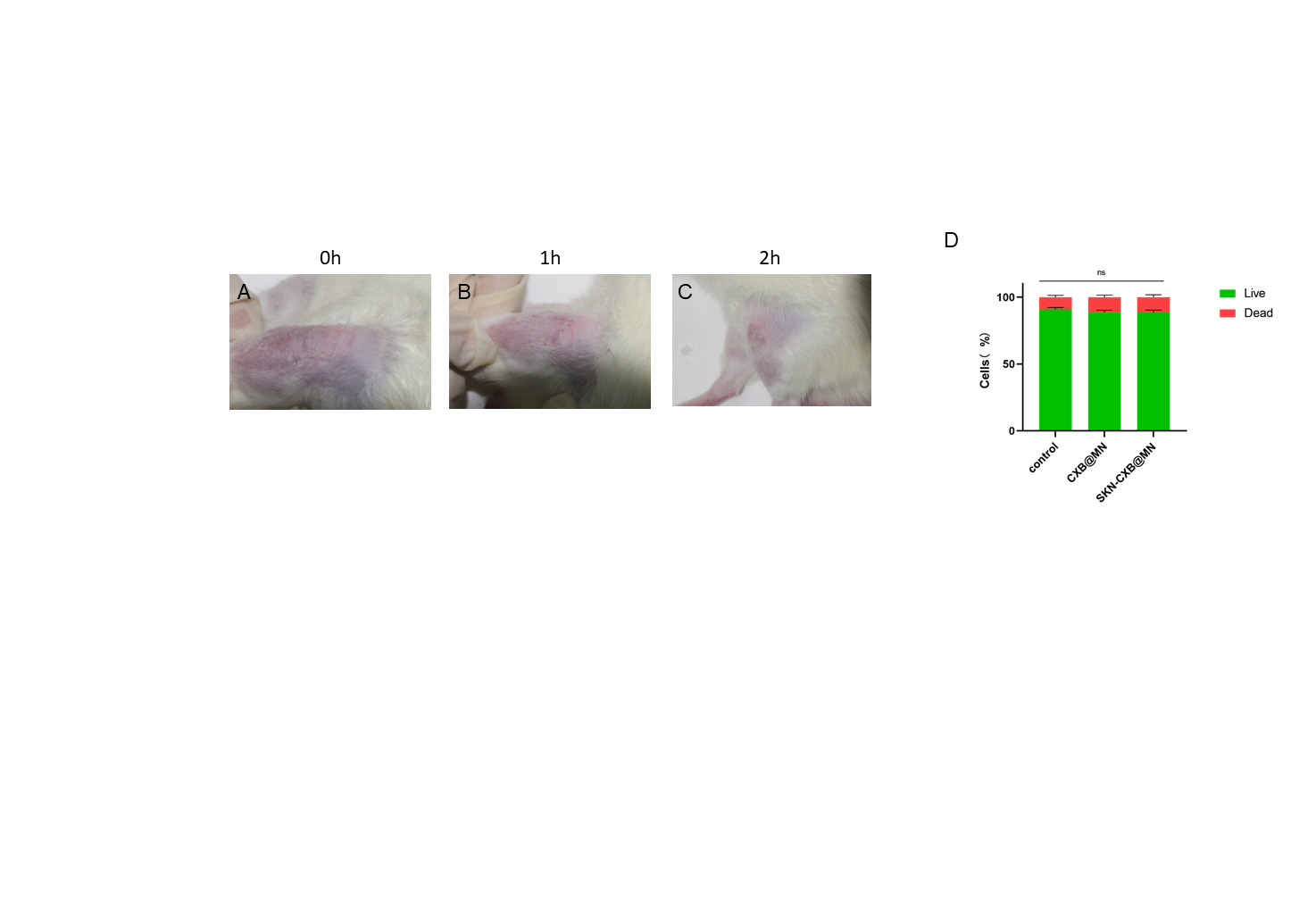
**

**Supplementary Figure 1.** Figure S1 (A-C) The image of skin after the removal of the MNs at 0, 1, and 2 h. (D) Quantitative analysis of Live/Dead staining.
